# 36 Juvenile idiopathic arthritis-associated uveitis: a study of 69 cases

**DOI:** 10.1093/rheumatology/keac496.032

**Published:** 2022-10-07

**Authors:** Djohra Hadef, Samy Slimani, Mohamed Choukri Khamari, Walid Mekaoussi, Alexandre Belot, Pierre Quartier

**Affiliations:** 1 Department of Pediatrics, University Hospital Center of Batna, Faculty of Medicine, Batna 2 University; 2 Atlas Clinic of Rheumatology; 3 Private Clinic of Rheumatology, Batna, Algeria; 4 Pediatric Nephrology, Rheumatology, Dermatology Unit, Hôpital Femme Mère Enfant, Hospices Civils de Lyon, National Referee Centre for Rheumatic and AutoImmune and Systemic diseases in children (RAISE), Lyon, France; 5 Pediatric Immunology-Hematology and Rheumatology Unit Necker Hospital, Assistance Publique Hôpitaux de Paris, National Referee Centre for Rheumatic and AutoImmune and Systemic Diseases in Children (RAISE), Paris, France

## Abstract

**Background:**

Juvenile Idiopathic Arthritis (JIA) is the most common rheumatic disease association with uveitis in children. Uveitis is a significant cause of visual morbidity in children with JIA. Geographical variations in the incidence of uveitis in JIA have been reported around the world.

**Objectives:**

The aim of this study was to determine the prevalence of JIA associated uveitis and its features in Batna -Algeria- and to compare the findings with other JIA populations worldwide.

**Methods:**

A multicentre retrospective descriptive study was conducted in Batna health centers (public and private sectors), over a seven-year period from January 2013 to December 2019, based on data collected on JIA patients. As public sector source, we referred to the department of pediatrics of the university hospital center (CHU Benflis Touhami Batna), and as private sector source, we referred to private adult rheumatologists based in Batna. The studied variables were: gender, age at the initial symptoms, age at diagnosis, JIA subtype based on International League of Associations for Rheumatology (ILAR) criteria, symptoms at onset, disease duration at the latest follow up, presence of uveitis, auto antibodies (antinuclear antibodies, Rheumatoid Factor and anti-CCP) pattern, joint imaging results, JIA medications, JIA status at the time of enrolment and the latest follow-up.

**Results:**

Sixty-nine cases of JIA were included. The female to male ratio was 1.8 :1. The median age at diagnosis was 9 years (range 1–16). At the latest follow-up, the median disease duration onset was 1 year (range 1–8 years). There were 34(49.3%) oligoarthritis, 9(13%) rheumatoid factor (RF) negative polyarticular arthritis, 8(11.6%) rheumatoid factor (RF) positive polyarticular arthritis, 6(8.7%) systemic onset JIA, 6(8.7%) enthesitis-related arthritis, 3 (4.3%) psoriatic arthritis, and 3(4.3%) undifferentiated arthritis. Nine patients (18.7%) were anti-nuclear antibody (ANA) positive, and 21 patients (30.4%) had indeterminate ANA status. Sixty-three patients (91.3%) have benefited from the slit lamp examination. Uveitis was found in 5 patients (7.2%). three of these patients presented with oligoarticular JIA and two presented Polyarticular JIA. There was male predominance (4 boys and 1 girl). All these patients were ANA negative.

**Conclusion:**

The observed phenotypic variabilities in JIA associated uveitis underscore the existence of true diversities in disease characteristics across geographical areas. Prospective multicentre studies are necessary to better identify the JIA associated uveitis peculiarities in our country.

**Disclosure of Interest:**

None declared

